# Association of antimüllerian hormone with polycystic ovarian syndrome phenotypes and pregnancy outcomes of in vitro fertilization cycles with fresh embryo transfer

**DOI:** 10.1186/s12884-022-04518-0

**Published:** 2022-03-02

**Authors:** Su Liu, Ling Hong, Meilan Mo, Shan Xiao, Xuejin Wang, Xinfeng Fan, Sainan Zhang, Lianghui Diao, Yong Zeng

**Affiliations:** 1 Shenzhen Key Laboratory of Reproductive Immunology for Peri-Implantation, Shenzhen Zhongshan Institute for Reproduction and Genetics, Fertility Center, Shenzhen Zhongshan Urology Hospital, Shenzhen, PR China; 2 Reproductive Medical Center, Renmin Hospital of Wuhan University & Hubei Clinic Research Center for Assisted Reproductive Technology and Embryonic Development, Wuhan, PR China

**Keywords:** Antimüllerian hormone, In vitro fertilization, Polycystic ovary syndrome, Pregnancy outcomes

## Abstract

**Objective:**

The current study was undertaken to investigate the relationship between antimüllerian hormone (AMH) and polycystic ovarian syndrome (PCOS) phenotypes and to determine whether AMH is associated with pregnancy outcomes in infertile women undergoing their first in vitro fertilization (IVF) treatment.

**Methods:**

We performed a retrospective cohort study of 2973 infertile women, including 418 women with PCOS undergoing their first IVF treatment at a private fertility center from January 2014 to March 2018. Women were stratified into three groups using cutoffs defined by the 25^th^ and 75^th^ percentiles of the serum AMH level: 746 women had AMH ≤ 2.25 ng/mL; 1486 women had AMH between 2.25 to 5.71 ng/mL; and 741 women had AMH > 5.71 ng/mL. Endocrine characteristics, PCOS phenotypes, stimulation outcomes, pregnancy outcomes were compared among these groups. When there were any statistical differences (*P* < 0.05) among the three groups, Bonferroni test was performed as post-hoc tests to determine where the statistical differences existed. To assess the relationships between AMH and pregnancy outcomes in total patients and PCOS patients, logistic regression analysis, adjusted for potential confounding covariates, were performed.

**Results:**

Women with high AMH had greater prevalence of hyperandrogenism (HA), polycystic ovarian morphology (PCOM) and amenorrhea than women with low or average AMH. The clinical pregnancy rate were significantly higher in the high-AMH group compared with low- and average-AMH groups (69.9% vs. 58.8% and 64.7% respectively; *P* < 0.001). The live birth rate was significantly lower in women with AMH ≤ 2.25 ng/mL compared with average- and high-AMH groups (47.6% vs. 55.2 and 59.5% respectively; *P* < 0.001). However, after controlling for maternal age, oocyte yield, as well as other confounders, AMH was no longer associated with a higher live birth rate (aOR 1.037, 95% CI 0.853–1.261, *P* = 0.717; aOR 1.099, 95% CI 0.858–1.408, *P* = 0.455, respectively) and clinical pregnancy rate (aOR 1.064, 95% CI 0.834–1.359, *P* = 0.617; aOR 1.181, 95% CI 0.875–1.595, *P* = 0.276, respectively). Moreover, pregnancy outcomes did not differ in PCOS women according to AMH quartiles.

**Conclusion:**

Increased AMH levels associated with PCOS severity and greater ovarian stimulation. However, AMH was not associated with clinical pregnancy rate and live birth rate after controlling for other confounders in women undergoing IVF. Thus, AMH should not be used to alter clinical decisions and exclude patients based on a low or even undetectable AMH value.

**Supplementary Information:**

The online version contains supplementary material available at 10.1186/s12884-022-04518-0.

## Introduction

The optimization and individualization of controlled ovarian hyperstimulation (COH) for in vitro fertilization (IVF) depends largely on the accurate prediction of ovarian response through patient characteristics and biomarkers. Antimüllerian hormone (AMH), also known as müllerian inhibiting substance, is a member of the transforming growth factor-β superfamily [1]. It is secreted primarily by the granulosa cells (GCs) of pre-antral and small antral follicles [2], and is well established as a predictor of ovarian reserve [3]. It acts as a follicular gatekeeper inhibiting initial follicle recruitment and follicle-stimulating hormone (FSH)-dependent growth and selection [4].

AMH has low intracycle and intercyle variability [5], and the dose–response relationship of AMH with ovarian response at COH is well established [6]. In addition to the association with quantitative response to ovarian stimulation, AMH may also be associated with qualitative assisted reproductive technology (ART) outcomes, such as clinical pregnancy and live birth rates independent of age [7]. However, the results were still controversial. Two meta-analyses examining the association of AMH with pregnancy outcomes in women undergoing IVF showed a weak association with implantation, pregnancy, and live birth, and that its predictive accuracy for these outcomes is poor [8].

A major factor contributing to the considerable variation between studies of the association of AMH with pregnancy outcomes is the heterogeneity in individual patient populations, stimulation protocols, AMH assay used, ultrasound techniques, as well as others. The predictive value of AMH for ART outcomes may be markedly different in specific subpopulations of infertility patients. Polycystic ovarian syndrome (PCOS) is the most common endocrine disorder affecting women of reproductive age, with an estimated prevalence of 5–8%. Serum AMH level appears to be related to the severity of PCOS [9], and may not reflect their ovarian reserve, thus confounding the association between AMH and IVF outcomes. Thus, to assess the predictive ability of AMH for IVF outcomes in specific subpopulations of infertility patients, we separately analyzed women with PCOS.

The main aim of this study was to investigate the relationship between AMH levels and PCOS manifestations as well as to evaluate the associations of AMH with clinical pregnancy and live birth rates in a large, unselected cohort of patients undergoing their first fresh IVF cycles and separately women fulfilling the diagnostic criteria of PCOS.

## Methods

### Subjects

**Fig. 1:**
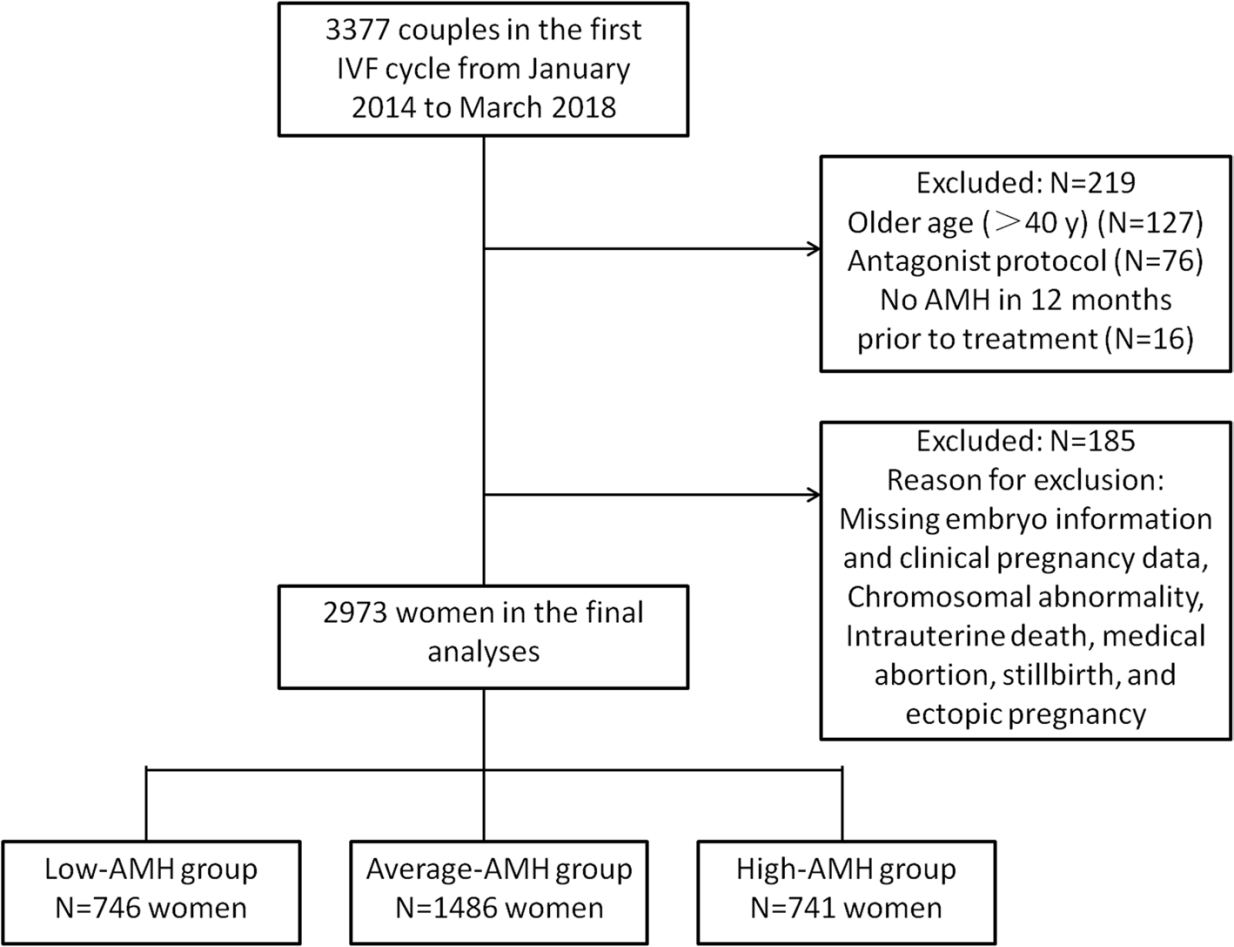
Flow chart showing the identification of the study population

This was a retrospective cohort study which included 2973 patients who presented to Shenzhen Zhongshan Urology Hospital for their first treatment between Jan 2014 and Mar 2018. Follow-up of pregnancies from the initial embryo transfer was completed in May 2019. All data entry, data management, and analyses were coordinated at our fertility center. We reviewed the medical charts of all patients seen during this time period (*n* = 3377) and excluded from this study all women aged > 40 years and those treated with gonadotropin-releasing hormone (GnRH)-antagonist COH protocols. We also excluded cycles missing embryo information and clinical pregnancy data and patients suffering from a chromosomal abnormality, intrauterine death, a medical abortion, stillbirth, or ectopic pregnancy. Patients were also excluded if their serum AMH measurements were not acquired within 12 months prior to their IVF treatment. The flowchart of patient selection was shown in Fig. 1. The womens’ medical histories were reviewed including the following parameters: age, body mass index (BMI), infertility history, serum FSH, luteinizing hormone (LH), AMH, total testosterone, thyroid-stimulating hormone (TSH), fasting glucose and fasting insulin.

As previous studies have described [10, 11], AMH values were stratified into three groups using cutoffs defined by 25^th^ and 75^th^ percentiles of the observations. Women were divided into 3 groups according to serum AMH level: low-AMH group (the 0-25^th^ percentage), average-AMH group (the 25^th^-75^th^ percentage) and high-AMH group (the 75^th^-100^th^ percentage).

As part of the infertility workup, all women underwent evaluation for PCOS. The diagnosis of PCOS was made when at least 2 of the following 3 criteria existed: Oligo- or amenorrhea, biochemical or clinical hyperandrogenism, and polycystic ovaries, according to the Rotterdam criteria [12]. Oligomenorrhea was defined as less than 8 cycles/year or menstrual interval > 35 days, whereas amenorrhoea was defined as absence of menstruation in the last 6 or more months. Hyperandrogenism included clinical and biochemical hyperandrogenism: the former was defined by the Ferriman-Galwey score more than 6 [13]; the latter was defined by a total testosterone (T) more than or equal to 0.481 ng/ml. The polycystic ovarian morphology (PCOM) was defined as ≥ 12 follicles in either ovary, measuring 2–9 mm in diameter and/or increased ovarian volume of each ovary > 10 ml on ultrasound scan [14]. No PCOS patients had evidence of congenital adrenal hyperplasia, Cushing’s syndrome and androgen-secreting tumours, non-classic adrenal hyperplasia, thyroid dysfunction, hyperprolactinemia, type 2 diabetes mellitus or cardiovascular disease. No subjects had received hormonal treatment or insulin-lowering agents in the previous quarter. Finally, the study population consisted of 418 women diagnosed with PCOS. This study was approved by the Institutional Review Board of Reproductive Research Ethics Committees of Shenzhen Zhongshan Urology Hospital (Approval number: SZZSECHU-F-2020042). All the patients signed a general informed consent form before their IVF treatment.

### Ovarian stimulation

All included patients received a standardized luteal phase down-regulation protocol with GnRH agonist protocol. The gonadotropin starting dose and the GnRH analogue were selected according to the ovarian response, as monitored on ultrasound for follicular tracking and blood sampling for estradiol levels performed every 1–3 days. Human chorionic gonadotropin (hCG) was administered to induce oocyte maturation when at least two follicles measured 17–18 mm. Oocytes retrieval was performed 36 h later. Embryo quality was evaluated by two experienced embryologists based on morphologic criteria. On day 3 or day 5 after fertilization, high-quality embryos were selected for fresh transfer.

### Measurement of outcomes

Pregnancy outcomes including implantation rate, clinical pregnancy rate, miscarriage rate and live birth rate were collected and measured. The implantation rate was calculated as the number of intrauterine gestational sacs divided by the number of embryos transferred. Live birth rate was calculated within the first fresh cycle, as the proportion of cycles resulting in live babies. A miscarriage was defined as the spontaneous demise of a pregnancy before the fetus reaches viability, which is from the time of conception until 28 weeks of gestation in China [15, 16], 24 weeks of gestation in European countries [17], or 22 weeks of gestation according to the international glossary on infertility and fertility care [18]. Thus, in this study, miscarriage included early miscarriage (pregnancy termination prior to 12 weeks of gestation) and late miscarriage (pregnancy termination within 12–28 weeks of gestation).

### Laboratory tests

All blood samples were collected in the morning after fasting for at least 8 hours and preferably on Days 2-5 of the spontaneous menstrual cycle in regularly menstruating women or during withdrawal bleeding in amenorrheic women. Specimens were stored at -70℃ until analysis was done within two weeks. All hormonal assays including TSH, estradiol (E_2_), LH, FSH, total T were carried out by chemiluminescence under Cobas e601 (Roche Diagnostics, Germany) using commercial kits, whereas plasma glucose and other biochemical parameters were assayed on Cobas c501 autoanalyzer (Roche Diagnostics, Germany). The minimal detectable limit for hormones was as follows: 0.02 ng/mL (total T), 5.0 pg/mL (E_2_), and 0.1 IU/L (FSH and LH), 0.01 μIU/mL (TSH). The inter-assay coefficients of variation were as follows: 4.5 % (total T), 4.9 % (E_2_), 4.5 % (FSH), 2.2 % (LH) and 1.8 % (TSH). The intra-assay coefficients of variation were as follows: 7.0 % (total T), 3.3 % (E_2_), 2.8 % (FSH), 1.2 % (LH) and 4.2 % (TSH). To investigate insulin sensitivity, the quantitative insulin sensitivity check index (QUICKI) was calculated. QUICKI=1/[log(I_0_)+log(G_0_)], where I_0_ is the fasting insulin, and G_0_ is the fasting glucose. QUICKI is a validated surrogate marker for insulin resistance and has good agreement with gold standard hyperinsulinaemic euglycaemic clamp [19].

Serum level of AMH, unrelated to the day of the menstrual cycle, was also analyzed by chemiluminescence immunoassay under Cobas e601 (Roche Diagnostics, Germany). Intra- and inter-assay coefficients of variation with serum control were approximately 1.0%-1.7% and 2.7%-3.3%, respectively. Minimum detection limit of the AMH test was 0.01 ng/mL, and maximum detection limit was 23 ng/mL. A new AMH test was done if IVF treatment was not commenced within 12 months, or the treatment was excluded from analysis.

### Statistical analysis

Continuous data with normal distribution were presented as the mean ± standard deviation (SD) and analyzed by analysis of variance (ANOVA), with Bonferroni test as post-hoc comparisons. The continuous variables without normal distribution were presented as median and interquartile range, and were analyzed by Kruskal–Wallis test, pairwise comparison. Categorical data were presented as frequencies with percentages, and were analyzed by Pearson’s χ^2^ test or Fisher's exact test. All significance tests were two tailed, and statistical significance was established as *P* < 0.05. All analyses were conducted using SPSS (version 23.0; SPSS Inc.). When there were any statistical differences (*P* < 0.05) among the three groups, Bonferroni test was performed as post-hoc tests to determine where the statistical differences existed. Data was missing in some cases completely at random.

To assess the relationships between AMH and pregnancy outcomes in total patients and PCOS patients, logistic regression analysis, adjusted for potential confounding covariates, were performed. For the pregnancy outcomes of total patients, the selected variables were those exhibit a *P*-value of < 0.05 in the univariate analysis. For the pregnancy outcomes of PCOS patients, the selection of variables includes clinical variables of known (i.e., age and number of embryos transferred), or suspected (i.e., AMH) prognostic importance for the outcome of interest, or other variables that exhibit a *P*-value of < 0.05 in the univariate analyses (i.e., endometrial thickness, number of fertilized oocytes, embryo type and quality). The number of oocytes retrieved was not included in the multiple regression analysis as it is highly correlated with both serum E_2_ level on the day of hCG trigger and number of fertilized occytes (Supplemental Table 1).

## Results

### Baseline characteristics, stimulation characteristics and outcomes according to AMH quartiles

**Fig. 2:**
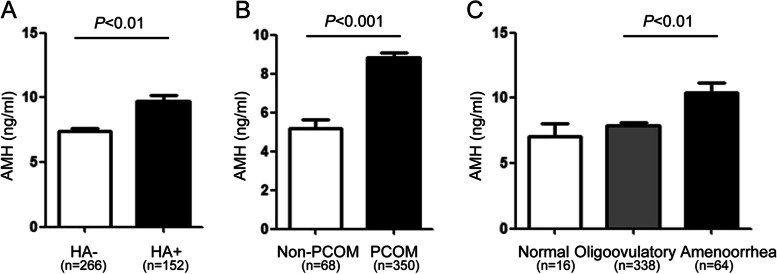
Comparison of AMH level (ng/mL) according to PCOS manifestations. **A**, hyperandrogenism, **B**, PCOM, **C**, menstrual regularity. AMH, antimüllerian hormone; HA, hyperandrogenism; PCOM, polycystic ovarian morphology. Mann–Whitney *U* test or Kruskal–Wallis test

2973 total women were tested for serum AMH level as part of their infertility workup between 2014–2018, and they were stratified into three groups using cutoffs defined by the 25^th^ and 75^th^ percentiles of the serum AMH level: 746 women (25.1%) had AMH ≤ 2.25 ng/mL; 1486 women (50.0%) had AMH between 2.25 to 5.71 ng/mL; and 741 women (24.9%) had AMH > 5.71 ng/mL. In total, 418 women (14.1%) in our study population were diagnosed as PCOS. The patients’ clinical and biochemical characteristics are shown in Table 1. Although no significant BMI differences were noted between groups, age was significantly greater in women with AMH ≤ 2.25 ng/mL compared with average- and high-AMH groups (33.0 vs. 31.0 and 31.0 years, respectively; both *P* < 0.001). LH was significantly higher in women with AMH > 5.71 ng/mL as compared with low- and average-AMH groups (6.20 vs. 3.87 and 4.62 IU/L, respectively; both *P* < 0.001). FSH was significantly lower in women with AMH > 5.71 ng/mL as compared with low- and average-AMH groups (5.83 vs. 6.62 and 6.44 IU/L, respectively; *P* = 0.020 and 0.037, respectively). Moreover, as AMH increased, the LH/FSH ratio significantly increased from 0.61 in the low-AMH group, to 0.92 in high-AMH group (*P* < 0.001). Similarly, as AMH increased, testosterone level demonstrated a gradual increase across AMH groups and was significantly higher in those with AMH > 5.71 ng/mL compared with low- and average-AMH groups (0.30 vs. 0.23 and 0.25 ng/mL, respectively; both *P* < 0.001). Overall, women in the AMH > 5.71 ng/mL group had significantly greater rate of hyperandrogenemia as compared with low-AMH and average-AMH groups (14.3% vs. 7.8% and 8.5%, respectively; *P* < 0.001). In addition, PCOS women with HA had significantly higher serum AMH level compared with PCOS women without HA (7.29 ng/mL, *n* = 152 vs. 6.77 ng/mL, *n* = 266, *P* = 0.005; see Fig. 2A). TSH, fasting glucose, fasting insulin and QUICKI did not differ among these groups.

**Table 1:** Biochemical and clinical characteristics of total patients according to AMH group

Maternal characteristics	Serum AMH, ng/mL			*P*-value
	** ≤ 2.25 (*n* = 746)**	**2.25–5.71 (*n* = 1486)**	** > 5.71 (*n* = 741)**	
**Female age (y)**	33.0 (30.0–36.0)^a,b^	31.0 (29.0–34.0)^b^	31.0 (28.0–33.0)	< 0.001^1^
**Female BMI (kg/m**^**2**^**)**	21.1 (19.5–22.8)	21.1 (19.5–23.1)	21.1 (19.5–23.4)	0.604^1^
**Type of infertility**				< 0.001^2^
Primary	337 (45.2%)^b^	750 (50.5%)	411 (55.5%)	
Secondary	409 (54.8%)	736 (49.5%)	330 (44.5%)	
**Duration of infertility (y)**	3.0 (1.5–5.0)	3.0 (2.0–5.0)	3.0 (2.0–5.0)	0.307^1^
**Cause of infertility**				
Uterine and tubal factor	287 (38.5%)^b^	559 (37.6%)^b^	210 (28.3%)	< 0.001^2^
Ovulatory disorders	4 (0.5%)^a,b^	51 (3.4%)^b^	159 (21.5%)	< 0.001^2^
Endometriosis	65 (8.7%)	109 (7.3%)	42 (5.7%)	0.077^2^
Male factor	113 (15.1%)	228 (15.3%)	103 (13.9%)	0.655^2^
Female and male factor	64 (8.6%)	158 (10.6%)	92 (12.4%)	0.055^2^
Unexplained	213 (28.6%)^b^	381 (25.6%)^b^	135 (18.2%)	< 0.001^2^
**D3 serum FSH level (IU/L)**	6.62 (5.05–7.82)^c^	6.44 (5.39–7.53)^c^	5.83 (4.22–8.34)	0.013^1^
**D3 serum LH level (IU/L)**	3.87 (2.85–5.16)^a,b^	4.62 (3.43–6.17)^b^	6.20 (5.34–7.21)	< 0.001^1^
**LH/FSH ratio**	0.61 (0.45–0.81)^a,b^	0.72 (0.55–0.95)^b^	0.92 (0.72–1.33)	< 0.001^1^
**Testosterone(ng/ml)**	0.23 (0.15–0.32)^a,b^	0.25 (0.18–0.33)^b^	0.30 (0.22–0.40)	< 0.001^1^
**TSH (μIU/mL)**	1.92 (1.40–2.69)	1.95 (1.38–2.69)	2.01 (1.44–3.01)	0.043^1^
**Fasting glucose (mmol/L)**	5.19 (4.85–5.67)	5.25 (4.95–5.56)	5.25 (4.94–5.63)	0.744^1^
**Fasting insulin (μU/mL)**	9.90 (6.60–17.50)	10.40 (7.28–15.65)	10.50 (7.30–15.35)	0.947^1^
**QUICKI**	0.336 ± 0.033	0.334 ± 0.032	0.334 ± 0.030	0.846^3^
**Hyperandrogenemia, %**	58 (7.8%)^b^	127 (8.5%)^b^	106 (14.3%)	< 0.001^2^
**Polycystic ovaries, %**	8 (1.1%)^a,b^	108 (7.3%)^b^	267 (36.0%)	< 0.001^2^
**Menstrual regularity**				< 0.001^2^
Regular periods, %	606 (81.2%)^a,b^	974 (65.5%)^b^	298 (40.2%)	
Oligomenorrhea, %	138 (18.5%)^a,b^	495 (33.3%)^b^	385 (52.0%)	
Amenorrhea, %	2 (0.3%)^b^	17 (1.1%)^b^	58 (7.8%)	
**PCOS diagnosis, %**	16 (2.1%)^a,b^	134 (9.0%)^b^	268 (36.2%)	< 0.001^2^
**Gonadotropin start dose(IU)**	300.0 (225.0–300.0)^a,b^	225.0 (150.0–300.0)^b^	150.0 (112.5–225.0)	< 0.001^1^
**Duration of gonadotropin stimulation (d)**	9.0 (8.0–11.0)	9.0 (8.0–10.0)	9.0 (8.0–10.0)	0.041^1^
**Total dose of gonadotropin used (IU)**	2700.0 (2287.5–3000.0)^a,b^	2400.0 (1837.5–2756.4)^b^	1800.0 (1350.0–2262.4)	< 0.001^1^
**Serum E**_**2**_** level (pg/mL) on hCG day**	1886.0 (1342.0–2523.5)^a,b^	2601.0 (1924.8–3765.8)^b^	2912.0 (2223.0–4134.5)	< 0.001^1^
**Endometrial thickness (mm) on hCG day**	11 (10–13)	11 (10–13)	11 (10–12)	0.530^1^
**No. of oocyte retrieved**	9 (7–12)^a,b^	12 (10–15)^b^	14 (11–16)	< 0.001^1^
**No. of oocyte fertilized**	7 (5–10)^a,b^	10 (7–13)^b^	11 (9–14)	< 0.001^1^
**No. of embryos transferred**	2.02 ± 0.61^a^	1.92 ± 0.55	1.95 ± 0.45	0.001^1^
**Cycles with different technologies**				0.056^2^
IVF	634 (85.0%)	1242 (83.6%)	648 (87.4%)	
ICSI	112 (15.0%)	244 (16.4%)	93 (12.6%)	
**Embryo type**				< 0.001^2^
Cleavage embryo	509 (68.2%)^a,b^	847 (57.0%)	457 (61.7%)	
Blastocyst	237 (31.8%)	639 (43.0%)	284 (38.3%)	
**Embryo quality**				0.003^2^
Cycle with high-quality embryos	701 (94.0%)^d^	1412 (95.0%)^d^	723 (97.6%)	
Cycles without high-quality embryos	45 (6.0%)	74 (5.0%)	18 (2.4%)	

*Note*: *BMI* Body mass index, *FSH* Follicle-stimulating hormone, *LH* Luteinizing hormone, *TSH* Thyroid-stimulating hormone, *QUICKI* Quantitative insulin sensitivity check index, *E*_*2*_ Estradiol, *IVF* in vitro fertilization, *ICSI* Intracytoplasmic sperm injection, *hCG* human chorionic gonadotropin

Values are numbers (percentages) of participants, median (interquartile range) or mean ± standard deviation

^1^Kruskal-Wallis test followed by a post-hoc pairwise comparison

^2^Chi-square test followed by Bonferroni post-hoc test

^3^One-way ANOVA followed by Bonferroni post-hoc test

^a^*P* < 0.001, statistically significant differences from average-AMH group

^b^*P* < 0.001, statistically significant differences from high-AMH group

^c^*P* < 0.05, statistically significant differences from high-AMH group

^d^*P* < 0.01, statistically significant differences from high-AMH group

The rate of PCOM was significantly greater in women with AMH > 5.71 ng/mL compared with low- and average-AMH groups (36.0% vs. 1.1% and 7.3%, respectively; *P* < 0.001). Similarly, PCOS diagnosis was significantly more prevalent in the high-AMH group compared with low-AMH group and average-AMH group (36.2% vs. 2.1% and 9.0% respectively; *P* < 0.001). Additionally, PCOS women with PCOM had significantly greater serum AMH levels compared with those without PCOM (7.60 ng/mL, *n* = 350 vs. 4.88 ng/mL, *n* = 68, *P* < 0.001; see Fig. 2B).

A significantly greater proportion of women with AMH ≤ 2.25 ng/mL had regular periods compared with women in average-AMH and high-AMH groups (81.2% vs. 65.5% and 40.2%, respectively; *P* < 0.001), whereas the proportion of women with amenorrhea was significantly greater in women with AMH > 5.71 ng/mL compared with low-AMH and average-AMH groups (7.8% vs. 0.3% and 1.1%, respectively; *P* < 0.001). PCOS women with amenorrhea demonstrated significantly greater serum AMH level than oligoovulatory women (8.95 ng/mL, *n* = 64 vs. 6.80 ng/mL, *n* = 338, *P* = 0.002; see Fig. 2C).

For the stimulation characteristics, the patients with AMH > 5.71 ng/mL had received a significantly lower dose of starting gonadotropin (150.0 vs. 300.0 and 225.0, respectively; both *P* < 0.001) and total gonadotropin (1800.0 vs. 2700.0 and 2400.0, respectively; both *P* < 0.001) compared with low- and average-AMH groups. There was no significant difference in the duration of gonadotropin stimulation and endometrial thickness before embryo transfer among these three groups. There was a significant between-group difference for the cycle stimulation outcomes, women with AMH > 5.71 ng/mL had a trend toward higher serum E_2_ level on the day of hCG trigger (2912.0 vs. 1886.0 and 2601.0 pg/mL, respectively; *P* < 0.001), more oocytes retrieved (14.0 vs. 9.0 and 12.0, respectively; *P* < 0.001) as well as higher number of fertilized oocytes (11.0 vs. 7.0 and 10.0, respectively; *P* < 0.001) compared with low- and average-AMH groups. More embryos were transferred in women with AMH ≤ 2.25 ng/mL compared with average- and high-AMH groups (2.02 vs. 1.92 and 1.95, respectively; *P* = 0.001). More cycles with cleavage stage embryos were found in women with AMH ≤ 2.25 ng/mL compared with average- and high-AMH groups (68.2% vs. 57.0% and 61.7%, respectively; *P* < 0.001). More high-quality embryos were transferred in women with AMH > 5.71 ng/mL compared with low- and average-AMH groups (97.6% vs. 94.0 and 95.0%, respectively; *P* = 0.003).

### Pregnancy outcomes according to AMH quartiles

The implantation rate and clinical pregnancy rate were significantly higher in the high-AMH group compared with the low- and average-AMH groups (0.53 vs. 0.42 and 0.50, 69.9% vs. 58.8% and 64.7%, respectively; both *P* < 0.001, see Table 2). The live birth rate was significantly lower in women with AMH ≤ 2.25 ng/mL compared with average- and high-AMH groups (47.6% vs. 55.2 and 59.5% respectively; *P* < 0.001). The miscarriage rate was significantly higher in low-AMH group compared with average- and high-AMH groups (18.0% vs. 12.9% vs. 12.7% respectively; *P* = 0.024). In addition, multiple pregnancies were more common among women with AMH > 5.71 ng/mL than among low- and average-AMH groups (34.6% vs. 24.3% and 30.2%, respectively; *P* = 0.007).

**Table 2:** Pregnancy outcomes of total patients according to AMH level

Outcomes	Serum AMH, ng/mL			*P*-value
	** ≤ 2.25 (*n* = 746)**	**2.25–5.71 (*n* = 1486)**	** > 5.71 (*n* = 741)**	
**Implantation rate**	0.42 ± 0.41^a,b^	0.50 ± 0.43	0.53 ± 0.42	< 0.001^1^
**Clinical pregnancy rate**	58.8% (439/746)^a,b^	64.7% (961/1486)^b^	69.9% (518/741)	< 0.001^2^
**Miscarriage rate**	18.0% (79/439)^c^	12.9% (124/961)	12.7% (66/518)	0.024^2^
Early miscarriage rate	13.4% (59/439)^d^	9.7% (93/961)	8.3% (43/518)	0.025^2^
Late miscarriage rate	4.6% (20/439)	3.2% (31/961)	4.4% (23/518)	0.353^2^
**Live birth rate**	47.6% (354/743)^a,b^	55.2% (814/1474)	59.5% (436/733)	< 0.001^2^
**No. of live babies delivered**				0.007^2^
1	75.7% (268/354)^e^	69.8% (568/814)	65.4% (285/436)	
≥ 2	24.3% (86/354)	30.2% (246/814)	34.6% (151/436)	

*Note*: Continuous variables: ^1^Kruskal-Wallis test followed by a post-hoc pairwise comparison. Categorical variables: ^2^Chi-square test followed by post-hoc Bonferroni correction

^a^*P* < 0.001, statistically significant differences from average-AMH group

^b^*P* < 0.001, statistically significant differences from high-AMH group

^c^*P* < 0.05, statistically significant differences from average-AMH group

^d^*P* < 0.05, statistically significant differences from high-AMH group

^e^*P* < 0.01, statistically significant differences from high-AMH group

Logistic regression analysis was performed to analyze the association between AMH and the pregnancy outcomes. As indicated in Table 3, more live births were positively correlated with higher AMH, younger age, less gonadotropin used, higher E_2_ levels and thicker endometrial thickness on the day of hCG trigger, more oocytes fertilized, more blastocysts and higher-quality embryos transferred in the univariate analysis. However, after correcting for the effects of all the above-mentioned confounders, AMH was no longer associated with a higher live birth rate (aOR 0.910, 95% CI 0.710–1.166, *P* = 0.455; aOR 0.943, 95% CI 0.776–1.148, *P* = 0.561, respectively). As for clinical pregnancy outcome, although AMH was significantly associated with clinical pregnancy on univariate analysis, AMH was not associated with clinical pregnancy after controlling for other confounders (aOR 0.843, 95% CI 0.653–1.089, *P* = 0.191; aOR 0.863, 95% CI 0.703–1.060, *P* = 0.160, respectively).

**Table 3:** Logistic regression analysis on the contribution of the potential predicting variables to live birth and clinical pregnancy outcomes of total patients

Variables	Live birth				Clinical pregnancy			
	**OR (95% CI)**	***P*-value**	**Adjusted OR** **(95% CI)**^**a**^	***P*-value**^**a**^	**OR (95% CI)**	***P*-value**	**Adjusted OR** **(95% CI)**^**a**^	***P*-value**^**a**^
**Group**								
Low-AMH	0.620 (0.504–0.762)	< 0.001	0.910 (0.710–1.166)	0.455	0.616 (0.497–0.763)	< 0.001	0.843 (0.653–1.089)	0.191
Average-AMH	0.840 (0.702–1.006)	0.057	0.943 (0.776–1.148)	0.561	0.788 (0.652–0.953)	0.014	0.863 (0.703–1.060)	0.160
High-AMH	Ref	Ref	Ref	Ref	Ref	Ref	Ref	Ref
**Female age (y)**	0.938 (0.921–0.956)	< 0.001	0.958 (0.938–0.978)	< 0.001	0.949 (0.930–0.968)	< 0.001	0.970 (0.950–0.991)	0.005
**Female BMI (kg/m**^**2**^**)**	1.001 (0.998–1.004)	0.553			1.001 (0.997–1.005)	0.590		
**Duration of infertility (y)**	0.980 (0.953–1.007)	0.148			0.987 (0.959–1.016)	0.383		
**Total dose of gonadotropin used (IU)**^**1**^	0.750 (0.677–0.831)	< 0.001	0.907 (0.804–1.023)	0.111	0.756 (0.680–0.840)	< 0.001	0.900 (0.796–1.018)	0.094
**Serum E**_**2**_** level (pg/mL) on hCG day**^**2**^	1.128 (1.074–1.184)	< 0.001	1.035 (0.976–1.099)	0.253	1.079 (1.026–1.135)	0.003	0.971 (0.913–1.032)	0.341
**Endometrial thickness (mm) on hCG day**	1.088 (1.053–1.124)	< 0.001	1.089 (1.052–1.126)	< 0.001	1.088 (1.052–1.126)	< 0.001	1.088 (1.050–1.127)	< 0.001
**No. of oocyte fertilized**	1.076 (1.056–1.098)	< 0.001	1.030 (1.004–1.056)	0.022	1.077 (1.055–1.099)	< 0.001	1.043 (1.016–1.070)	0.002
**No. of embryos transferred**	0.891 (0.780–1.018)	0.089			0.907 (0.790–1.042)	0.168		
**Embryo type**								
Cleavage embryo	Ref	Ref	Ref	Ref	Ref	Ref	Ref	Ref
Blastocyst	2.126 (1.824–2.477)	< 0.001	1.851 (1.567–2.187)	< 0.001	2.095 (1.782–2.462)	< 0.001	1.839 (1.543–2.193)	< 0.001
**Embryo quality**								
Cycle with high-quality embryos	Ref	Ref	Ref	Ref	Ref	Ref	Ref	Ref
Cycles without high-quality embryos	0.309 (0.211–0.453)	< 0.001	0.313 (0.211–0.465)	< 0.001	0.339 (0.239–0.482)	< 0.001	0.341 (0.236–0.491)	< 0.001

*Note*: All the variables inputted in the model were shown in Table 3. *BMI* Body mass index, *CI* Confidence interval, *OR* Odds ratio, *E*_*2*_ Estradiol, *hCG* human chorionic gonadotropin, REF Reference, ^1^ per 1000 IU increased, ^2^ per 1000 pg/mL increased

^a^Adjusted for maternal age, total dose of gonadotropin used, serum E_2_ level and endometrial thickness on the day of ovulatory dose of hCG, number of fertilization, embryo type and embryo quality

### Baseline characteristics and pregnancy outcomes of PCOS women according to AMH quartiles

Patients with PCOS were further divided into 3 groups according to the 25^th^ and 75^th^ percentile of the serum AMH level: AMH ≤ 4.91 ng/mL (*n* = 106), AMH between 4.91 and 10.88 ng/mL (*n* = 208) and AMH > 10.88 ng/mL (*n* = 104). Biochemical and cycle characteristics were compared among these three groups and are summarized in Supplemental Table 2. Age was not significantly different among the three groups. BMI was significantly lower in women with AMH > 10.88 ng/mL compared with low- and average-AMH groups (21.37 vs. 22.49 and 22.64 kg/m^2^, respectively; *P* = 0.001). LH was significantly higher in the high-AMH group compared with low- and average-AMH groups (8.97 vs. 4.92 and 6.27 IU/L, respectively; *P* < 0.001). The patients with AMH > 10.88 ng/mL had received a significantly lower dose of starting gonadotropin (112.5 vs. 150.0 and 150.0, respectively; *P* < 0.001) and total gonadotropin (1350.0 vs. 2025.0 and 1687.5, respectively; *P* < 0.001) compared with low- and average-AMH groups. Women with AMH > 10.88 ng/mL had a trend toward higher serum E_2_ level on the day of hCG trigger compared with low- and average-AMH groups (2897.5 vs. 2434.5 and 2568.5 pg/mL, respectively; *P* = 0.037). As for cycle stimulation outcomes, the number of retrieved oocytes and fertilized oocytes were comparable among the three groups. Although there were more high-quality embryos transferred in the average-AMH group compared with low-AMH group, the pregnancy outcomes including implantation rate, clinical pregnancy rate, miscarriage rate, live birth rate and multiple pregnancy rate were all similar among these three groups, indicating AMH was not associated with pregnancy outcomes in PCOS patients (Table 4).

**Table 4:** Pregnancy outcomes of PCOS patients according to AMH level

Outcomes	Serum AMH, ng/mL			*P*-value
	** ≤ 4.91 (*n* = 106)**	**4.91–10.88 (*n* = 208)**	** > 10.88 (*n* = 104)**	
**Implantation rate**	0.56 ± 0.45	0.55 ± 0.42	0.52 ± 0.44	0.812^1^
**Clinical pregnancy rate**	69.8% (74/106)	73.1% (152/208)	67.3% (70/104)	0.553^2^
**Miscarriage rate**	8.1% (6/74)	19.1% (29/152)	17.1% (12/70)	0.101^2^
Early miscarriage rate	5.4% (4/74)	11.2% (17/152)	10.0% (7/70)	0.373^2^
Late miscarriage rate	2.7% (2/74)	7.9% (12/152)	7.1% (5/70)	0.313^3^
**Live birth rate**	62.3% (66/106)	56.8% (117/206)	53.4% (55/103)	0.429^2^
**No. of live babies delivered**				0.351^2^
1	66.7% (44/66)	69.2% (81/117)	58.2% (32/55)	
≥ 2	33.3% (22/66)	30.8% (36/117)	41.8% (23/55)	

*Note*: Continuous variables: ^1^Kruskal-Wallis test. Categorical variables: ^2^Chi-square test; ^3^Fisher’s exact test

Logistic regression analysis was also performed to analyze the association between AMH and the pregnancy outcomes in PCOS patients. After controlling for maternal age, embryo type and quality, as well as other confounders, AMH was not associated with a higher live birth rate (aOR 1.370, 95% CI 0.750–2.502, *P* = 0.306; aOR 1.045, 95% CI 0.630–1.734, *P* = 0.863, respectively) (Table 5). Similarly, AMH was not associated with clinical pregnancy after controlling for other confounders (aOR 1.016, 95% CI 0.544–1.901, *P* = 0.959; aOR 1.225, 95% CI 0.717–2.093, *P* = 0.459, respectively).

**Table 5:** Logistic regression analysis on the contribution of the potential predicting variables to live birth and clinical pregnancy outcomes of PCOS patients

Variables	Live birth				Clinical pregnancy			
	**OR (95% CI)**	***P*-value**	**Adjusted OR** **(95% CI)**^**a**^	***P*-value**^**a**^	**OR (95% CI)**	***P*-value**	**Adjusted OR** **(95% CI)**^**a**^	***P*-value**^**a**^
**Group**								
Low-AMH	1.440 (0.830–2.500)	0.195	1.370 (0.750–2.502)	0.306	1.123 (0.627–2.012)	0.696	1.016 (0.544–1.901)	0.959
Average-AMH	1.147 (0.713–1.845)	0.571	1.045 (0.630–1.734)	0.863	1.318 (0.790–2.199)	0.290	1.225 (0.717–2.093)	0.459
High-AMH	Ref	Ref	Ref	Ref	Ref	Ref	Ref	Ref
**Female age (y)**	0.955 (0.904–1.009)	0.098	0.928 (0.872–0.986)	0.017	0.957 (0.902–1.016)	0.149	0.934 (0.875–0.998)	0.043
**Female BMI (kg/m**^**2**^**)**	1.002 (0.996–1.007)	0.583			1.003 (0.980–1.026)	0.809		
**Duration of infertility (y)**	0.985 (0.912–1.063)	0.696			1.000 (0.920–1.087)	0.999		
**Total dose of gonadotropin used (IU)**^**1**^	1.035 (0.802–1.335)	0.794			1.100 (0.828–1.460)	0.511		
**Serum E**_**2**_** level (pg/mL) on hCG day**^**2**^	1.212 (1.066–1.379)	0.003			1.103 (0.963–1.263)	0.158		
**Endometrial thickness (mm) on hCG day**	1.104 (1.008–1.209)	0.034	1.094 (0.990–1.208)	0.077	1.078 (0.977–1.191)	0.135	1.064 (0.958–1.183)	0.248
**No. of oocyte fertilized**	1.054 (1.002–1.108)	0.042	1.014 (0.960–1.071)	0.626	1.057 (1.001–1.117)	0.046	1.011 (0.954–1.071)	0.711
**No. of embryos transferred**	0.907 (0.591–1.391)	0.654	1.916 (1.094–3.358)	0.023	0.860 (0.538–1.373)	0.527	1.871 (1.007–3.476)	0.048
**Embryo type**								
Cleavage embryo	Ref	Ref	Ref	Ref	Ref	Ref	Ref	Ref
Blastocyst	2.747 (1.812–4.164)	< 0.001	3.488 (2.023–6.013)	< 0.001	2.790 (1.742–4.468)	< 0.001	3.659 (1.970–6.795)	< 0.001
**Embryo quality**								
Cycle with high-quality embryos	Ref	Ref	Ref	Ref	Ref	Ref	Ref	Ref
Cycles without high-quality embryos	0.158 (0.034–0.742)	0.019	0.172 (0.035–0.841)	0.030	0.332 (0.099–1.110)	0.073	0.423 (0.120–1.488)	0.180

*Note*: All the variables inputted in the model were shown in Table 5. *BMI* Body mass index, *CI* Confidence interval, *OR* Odds ratio, *E*_*2*_ Estradiol, *hCG* human chorionic gonadotropin, *Ref* Reference, ^1^ per 1000 IU increased, ^2^ per 1000 pg/mL increased

^a^Adjusted for maternal age, endometrial thickness on the day of ovulatory dose of hCG, number of fertilization, number of embryos transferred, embryo type and embryo quality

## Discussion

The present study demonstrated that all 3 PCOS diagnostic hallmarks, namely hyperandrogenism, PCOM, and oligo/amenorrhea, were associated with high AMH levels. Clinical pregnancy rate and live birth rate were significantly higher in women with high AMH levels. However, AMH was no longer associated with a higher live birth rate and clinical pregnancy rate after controlling for maternal age, oocyte yield, as well as other confounders. Moreover, pregnancy outcomes did not differ in PCOS women according to AMH quartiles.

In our study, women with high AMH level had greater prevalence of hyperandrogenism, PCOM, oligo/amenorrhea and PCOS, consistent with several earlier studies [20]. Our results suggest that increased AMH levels are associated with PCOS severity. Studies have shown that androgens play a role in stimulating early (FSH independent) stages of follicular growth [21]. AMH, in turn, could inhibit granulosa cell FSH-stimulated aromatase expression [22], resulting in the elevation of intraovarian androgens [23]. Moreover, our results showed that AMH levels were associated with menstrual cycle status, which was consistent with previous study showing that AMH had strong diagnostic ability for amenorrhea by receiver operating characteristic (ROC) curve analysis [20]. Similarly, Pigny et al. showed that AMH levels were related to menstrual disorder severity in PCOS women [24]. AMH has an inhibitory effect on antral follicular growth through inhibition of FSH responsiveness and aromatase expression [25]. AMH was shown to be highest in small antral follicles, which becomes very low or undetectable in follicles > 10 mm, suggesting that the cessation of AMH is critical for selection of the dominant follicle. Pigny et al. have reported that AMH is tightly related to the 2–5 mm follicular number in PCOS, which was associated with the severity of the menstrual disorder, being highest in women with amenorrhea [26].

In our study, women with increased AMH levels showed a trend toward increased number of oocytes retrieved, which is consistent with previous studies showing that AMH is an excellent predictor of ovarian response to stimulation [27]. Additionally, we observed higher clinical pregnancy and live birth rates in high-AMH group compared with the other two groups among total patients. However, AMH was no longer associated with pregnancy outcomes after adjustment for the following risk factors: maternal age, total dose of gonadotropin used, serum E_2_ level and endometrial thickness on the day of ovulatory dose of hCG, number of fertilized oocytes, embryo type and embryo quality. Consistent with our results, a recent meta-analysis of 19 studies suggested that the predictive accuracy of AMH on implantation and clinical pregnancy is limited [28]. The observed differences in pregnancy outcomes may be attributable by the greater availability of oocytes and embryos in high-AMH group. Consistently, although previous studies have shown an association between AMH and live birth after ART, additional statistical analyses suggested this association may be mediated and explained by the relationship between AMH and oocyte yield [29]. However, for the PCOS patients, their oocyte yield was not increased with increasing AMH value, which may disrupt the association between AMH and their pregnancy outcomes. Additionally, hyperandrogenemia accompanied with higher AMH is associated with decreased oocyte developmental competence, which may also explain the similar pregnancy outcomes of PCOS patients between high-AMH group and other groups[30]. Moreover, there is a possibility of impaired endometrial receptivity since high estradiol levels [31] often reached among the women with high response, with a premature induction of progesterone receptors resulting in an advanced endometrium [32], which may affect embryo implantation and confound the association of AMH with pregnancy outcomes. Overall, our results show that AMH was not associated with qualitative outcomes of ART such as clinical pregnancy and live birth, suggesting other factors may affect pregnancy outcomes.

Interestingly, age remains significant in multivariant analyses, suggesting that age may reflect another aspect of women’s fertility not covered by ovarian reserve test. The reason may be the age-related deterioration of oocyte competence in advanced-aged women [33], as the impairment of mitochondrial function [34] and the high production of reactive oxygen species (ROS) [35] were reported to be related with aged granulosa cells. The increased mitochondrial DNA instability and decreased mitochondrial biogenesis occur with aging could induce DNA damage in oocytes, and cause disassembly of oocyte spindles, as a consequence, decreases the oocyte quality [34]. In this study, age was more important than AMH level in the assessment of pregnancy outcomes among total patients.

There have been conflicting results on the association of serum AMH and pregnancy outcomes. The heterogeneity may be caused by differences in study populations, different stimulating protocols, and different AMH cut-off points. In an attempt to minimize heterogeneity originating from different study populations, separate analyses were performed on women with PCOS. In our analysis, AMH displayed no association with pregnancy outcomes in women with PCOS. This result could be explained by the similar number of retrieved oocytes among PCOS women with different AMH levels. PCOS is characterized by elevated AMH levels, which was also associated with all of the three clinical diagnostic hallmarks of PCOS. Some studies have shown that serum AMH have strong predictive accuracy for PCOS and suggested its incorporation as a diagnostic criterion for this disease [36]. Importantly, increased AMH level in PCOS is due largely to increased AMH production by individual follicles rather than increased follicle number [25]. This strong association of AMH levels with PCOS severity and limited association with follicle number may confound an association between AMH and ovarian reserve and quality, thus explaining the poor relationship between AMH and pregnancy outcome in women PCOS.

The strength of this study are a homogenous population of women undergoing their first IVF/ICSI treatment in one fertility center with very uniform treatment practices. Well-documented data allowed us to control the results with other confounding factors. The sub analysis of PCOS population is also a strength of this study. A limitation of our study include its retrospective and non-randomized design. Second, as the cases of ectopic pregnancies, intrauterine death, stillbirth, and medical abortions were few, they were not included in the main analysis, which may have increased the observed live birth rate. Third, analysis of the association between AMH and pregnancy outcomes was limited to the first fresh IVF cycle, without assessing the rates of clinical pregnancy and live birth in frozen embryo transfer cycles. Finally, the small sample size of PCOS patients is also an inherent weakness of this study.

## Conclusions

In conclusion, this study demonstrates that AMH associated with PCOS severity and were associated with greater ovarian stimulation. However, AMH was not associated with clinical pregnancy and live birth rates after controlling for other confounders in women undergoing IVF. Thus, AMH should not be used to alter clinical decisions and exclude patients based on a low or even undetectable AMH value. Our results may have clinical value on counseling and ultimately give comfort and hope to patients with low AMH, which is also valuable when assessing the overall effectiveness of IVF/ICSI treatment.

## Supplementary Information

**Additional file 1.**

## Data Availability

The datasets used and/or analyzed during the current study are available from the corresponding author on reasonable request.

## Abbreviations

AMH: Antimüllerian hormone
PCOS: Polycystic ovarian syndrome
IVF: In vitro fertilization
HA: Hyperandrogenism
PCOM: Polycystic ovarian morphology
COH: Controlled ovarian hyperstimulation
GC: Granulosa cell
FSH: Follicle-stimulating hormone
ART: Assisted reproductive technology
GnRH: Gonadotropin-releasing hormone
hCG: Human chorionic gonadotropin
T: Testosterone
TSH: Thyroid-stimulating hormone
E_2_: Estradiol
LH: Luteinizing hormone
QUICKI: Quantitative insulin sensitivity check index
BMI: Body mass index
ICSI: Intracytoplasmic sperm injection
