# Supplementary material for: Association of antimüllerian hormone with polycystic ovarian syndrome phenotypes and pregnancy outcomes of in vitro fertilization cycles with fresh embryo transfer
Source: BMC Pregnancy Childbirth. 2022 Mar 2;22:171. doi: 10.1186/s12884-022-04518-0 (PMC8892693; doi:10.1186/s12884-022-04518-0)
Supplement: Supplementary file 1 — Additional file 1. [file 12884_2022_4518_MOESM1_ESM.docx]

**SUPPLEMENTAL TABLE 1.**

Correlations between factors in the logistic models.

|  | | AMH level | Maternal age | Maternal BMI | Infertility factor | Infertility duration | GN start dose (IU) | GN duration | GN dose (IU) | E_2_ on hCG day | EMT on hCG day | No. of oocyte retrieved | No. of fertilized occytes | No. of embryo transferred | Technology | Embryo type | Embryo quality |
| --- | --- | --- | --- | --- | --- | --- | --- | --- | --- | --- | --- | --- | --- | --- | --- | --- | --- |
| AMH level | Pearson correlation | 1 | -.181^**^ | .028 | -.074^**^ | .003 | -.507^**^ | .036^*^ | -.406^**^ | .301^**^ | .002 | .360^**^ | .332^**^ | -.042^*^ | -.024 | .047^*^ | -.061^**^ |
|  | Sig. (2-tailed) |  | .000 | .133 | .000 | .880 | .000 | .047 | .000 | .000 | .912 | .000 | .000 | .021 | .187 | .010 | .001 |
|  | No. | 2973 | 2973 | 2973 | 2973 | 2973 | 2973 | 2973 | 2973 | 2973 | 2973 | 2973 | 2973 | 2973 | 2973 | 2973 | 2973 |
| Maternal age | Pearson correlation | -.181^**^ | 1 | .015 | .235^**^ | .193^**^ | .427^**^ | .011 | .311^**^ | -.105^**^ | -.068^**^ | -.142^**^ | -.111^**^ | .328^**^ | -.038^*^ | -.121^**^ | -.023 |
|  | Sig. (2-tailed) | .000 |  | .421 | .000 | .000 | .000 | .549 | .000 | .000 | .000 | .000 | .000 | .000 | .036 | .000 | .213 |
|  | No. | 2973 | 2973 | 2973 | 2973 | 2973 | 2973 | 2973 | 2973 | 2973 | 2973 | 2973 | 2973 | 2973 | 2973 | 2973 | 2973 |
| Maternal BMI | Pearson correlation | .028 | .015 | 1 | -.008 | -.019 | -.013 | .003 | -.006 | -.016 | .000 | .004 | .004 | .006 | -.010 | -.020 | -.008 |
|  | Sig. (2-tailed) | .133 | .421 |  | .681 | .291 | .487 | .865 | .730 | .396 | .981 | .817 | .819 | .733 | .573 | .280 | .654 |
|  | No. | 2973 | 2973 | 2973 | 2973 | 2973 | 2973 | 2973 | 2973 | 2973 | 2973 | 2973 | 2973 | 2973 | 2973 | 2973 | 2973 |
| Infertility factor | Pearson correlation | -.074^**^ | .235^**^ | -.008 | 1 | -.106^**^ | .093^**^ | -.010 | .061^**^ | -.040^*^ | -.111^**^ | -.031 | .025 | .016 | -.101^**^ | .014 | -.021 |
|  | Sig. (2-tailed) | .000 | .000 | .681 |  | .000 | .000 | .586 | .001 | .031 | .000 | .087 | .173 | .392 | .000 | .446 | .242 |
|  | No. | 2973 | 2973 | 2973 | 2973 | 2973 | 2973 | 2973 | 2973 | 2973 | 2973 | 2973 | 2973 | 2973 | 2973 | 2973 | 2973 |
| Infertility duration | Pearson correlation | .003 | .193^**^ | -.019 | -.106^**^ | 1 | .055^**^ | .068^**^ | .092^**^ | -.033 | .003 | -.048^**^ | -.061^**^ | .107^**^ | .016 | -.059^**^ | -.008 |
|  | Sig. (2-tailed) | .880 | .000 | .291 | .000 |  | .003 | .000 | .000 | .072 | .866 | .009 | .001 | .000 | .390 | .001 | .648 |
|  | No. | 2973 | 2973 | 2973 | 2973 | 2973 | 2973 | 2973 | 2973 | 2973 | 2973 | 2973 | 2973 | 2973 | 2973 | 2973 | 2973 |
| GN start dose (IU) | Pearson correlation | -.507^**^ | .427^**^ | -.013 | .093^**^ | .055^**^ | 1 | -.072^**^ | .708^**^ | -.219^**^ | -.025 | -.334^**^ | -.277^**^ | .196^**^ | -.010 | -.152^**^ | .045^*^ |
|  | Sig. (2-tailed) | .000 | .000 | .487 | .000 | .003 |  | .000 | .000 | .000 | .167 | .000 | .000 | .000 | .571 | .000 | .015 |
|  | No. | 2973 | 2973 | 2973 | 2973 | 2973 | 2973 | 2973 | 2973 | 2973 | 2973 | 2973 | 2973 | 2973 | 2973 | 2973 | 2973 |
| GN duration | Pearson correlation | .036^*^ | .011 | .003 | -.010 | .068^**^ | -.072^**^ | 1 | .546^**^ | .120^**^ | -.015 | .023 | .055^**^ | -.007 | -.019 | -.006 | .033 |
|  | Sig. (2-tailed) | .047 | .549 | .865 | .586 | .000 | .000 |  | .000 | .000 | .406 | .208 | .003 | .685 | .306 | .744 | .076 |
|  | No. | 2973 | 2973 | 2973 | 2973 | 2973 | 2973 | 2973 | 2973 | 2973 | 2973 | 2973 | 2973 | 2973 | 2973 | 2973 | 2973 |
| GN dose (IU) | Pearson correlation | -.406^**^ | .311^**^ | -.006 | .061^**^ | .092^**^ | .708^**^ | .546^**^ | 1 | -.121^**^ | -.020 | -.249^**^ | -.190^**^ | .127^**^ | -.015 | -.102^**^ | .068^**^ |
|  | Sig. (2-tailed) | .000 | .000 | .730 | .001 | .000 | .000 | .000 |  | .000 | .281 | .000 | .000 | .000 | .415 | .000 | .000 |
|  | No. | 2973 | 2973 | 2973 | 2973 | 2973 | 2973 | 2973 | 2973 | 2973 | 2973 | 2973 | 2973 | 2973 | 2973 | 2973 | 2973 |
| E_2_ on hCG day | Pearson correlation | .301^**^ | -.105^**^ | -.016 | -.040^*^ | -.033 | -.219^**^ | .120^**^ | -.121^**^ | 1 | -.049^**^ | **.562^**^** | .525^**^ | -.087^**^ | .057^**^ | .178^**^ | -.027 |
|  | Sig. (2-tailed) | .000 | .000 | .396 | .031 | .072 | .000 | .000 | .000 |  | .007 | .000 | .000 | .000 | .002 | .000 | .146 |
|  | No. | 2973 | 2973 | 2973 | 2973 | 2973 | 2973 | 2973 | 2973 | 2973 | 2973 | 2973 | 2973 | 2973 | 2973 | 2973 | 2973 |
| EMT | Pearson correlation | .002 | -.068^**^ | .000 | -.111^**^ | .003 | -.025 | -.015 | -.020 | -.049^**^ | 1 | -.009 | -.018 | -.017 | -.020 | .017 | -.004 |
|  | Sig. (2-tailed) | .912 | .000 | .981 | .000 | .866 | .167 | .406 | .281 | .007 |  | .618 | .327 | .368 | .271 | .365 | .836 |
|  | No. | 2973 | 2973 | 2973 | 2973 | 2973 | 2973 | 2973 | 2973 | 2973 | 2973 | 2973 | 2973 | 2973 | 2973 | 2973 | 2973 |
| No. of oocyte retrieved | Pearson correlation | .360^**^ | -.142^**^ | .004 | -.031 | -.048^**^ | -.334^**^ | .023 | -.249^**^ | **.562^**^** | -.009 | 1 | **.861^**^** | -.145^**^ | .065^**^ | .289^**^ | -.043^*^ |
|  | Sig. (2-tailed) | .000 | .000 | .817 | .087 | .009 | .000 | .208 | .000 | .000 | .618 |  | .000 | .000 | .000 | .000 | .020 |
|  | No. | 2973 | 2973 | 2973 | 2973 | 2973 | 2973 | 2973 | 2973 | 2973 | 2973 | 2973 | 2973 | 2973 | 2973 | 2973 | 2973 |
| No. of fertilized occytes | Pearson correlation | .332^**^ | -.111^**^ | .004 | .025 | -.061^**^ | -.277^**^ | .055^**^ | -.190^**^ | .525^**^ | -.018 | **.861^**^** | 1 | -.133^**^ | -.012 | .344^**^ | -.062^**^ |
|  | Sig. (2-tailed) | .000 | .000 | .819 | .173 | .001 | .000 | .003 | .000 | .000 | .327 | .000 |  | .000 | .526 | .000 | .001 |
|  | No. | 2973 | 2973 | 2973 | 2973 | 2973 | 2973 | 2973 | 2973 | 2973 | 2973 | 2973 | 2973 | 2973 | 2973 | 2973 | 2973 |
| No. of embryo transferred | Pearson correlation | -.042^*^ | .328^**^ | .006 | .016 | .107^**^ | .196^**^ | -.007 | .127^**^ | -.087^**^ | -.017 | -.145^**^ | -.133^**^ | 1 | -.004 | -.547^**^ | -.082^**^ |
|  | Sig. (2-tailed) | .021 | .000 | .733 | .392 | .000 | .000 | .685 | .000 | .000 | .368 | .000 | .000 |  | .825 | .000 | .000 |
|  | No. | 2973 | 2973 | 2973 | 2973 | 2973 | 2973 | 2973 | 2973 | 2973 | 2973 | 2973 | 2973 | 2973 | 2973 | 2973 | 2973 |
| Technology | Pearson correlation | -.024 | -.038^*^ | -.010 | -.101^**^ | .016 | -.010 | -.019 | -.015 | .057^**^ | -.020 | .065^**^ | -.012 | -.004 | 1 | .001 | -.003 |
|  | Sig. (2-tailed) | .187 | .036 | .573 | .000 | .390 | .571 | .306 | .415 | .002 | .271 | .000 | .526 | .825 |  | .976 | .866 |
|  | No. | 2973 | 2973 | 2973 | 2973 | 2973 | 2973 | 2973 | 2973 | 2973 | 2973 | 2973 | 2973 | 2973 | 2973 | 2973 | 2973 |
| Embryo type | Pearson correlation | .047^*^ | -.121^**^ | -.020 | .014 | -.059^**^ | -.152^**^ | -.006 | -.102^**^ | .178^**^ | .017 | .289^**^ | .344^**^ | -.547^**^ | .001 | 1 | -.008 |
|  | Sig. (2-tailed) | .010 | .000 | .280 | .446 | .001 | .000 | .744 | .000 | .000 | .365 | .000 | .000 | .000 | .976 |  | .664 |
|  | No. | 2973 | 2973 | 2973 | 2973 | 2973 | 2973 | 2973 | 2973 | 2973 | 2973 | 2973 | 2973 | 2973 | 2973 | 2973 | 2973 |
| Embryo quality | Pearson correlation | -.061^**^ | -.023 | -.008 | -.021 | -.008 | .045^*^ | .033 | .068^**^ | -.027 | -.004 | -.043^*^ | -.062^**^ | -.082^**^ | -.003 | -.008 | 1 |
|  | Sig. (2-tailed) | .001 | .213 | .654 | .242 | .648 | .015 | .076 | .000 | .146 | .836 | .020 | .001 | .000 | .866 | .664 |  |
|  | No. | 2973 | 2973 | 2973 | 2973 | 2973 | 2973 | 2973 | 2973 | 2973 | 2973 | 2973 | 2973 | 2973 | 2973 | 2973 | 2973 |

Note: * *P* < 0.05; ** *P* < 0.01.

**SUPPLEMENTAL TABLE 2.**

Biochemical and clinical characteristics of PCOS women according to AMH group.

| **Maternal characteristics** | **Serum AMH, ng/mL** | | | ***P*-value** |
| --- | --- | --- | --- | --- |
|  | **≤4.91 (n=106)** | **4.91-10.88 (n=208)** | **＞10.88 (n=104)** |  |
| **Female age (y)** | 31.0 (28.0-33.0) | 30.0 (28.0-33.0) | 30.0 (27.0-32.0) | 0.076^1^ |
| **Female BMI (kg/m^2^)** | 22.49 (20.46-25.85)^a^ | 22.64 (20.82-25.42)^b^ | 21.37 (19.36-23.81) | 0.001^1^ |
| **Type of infertility** |  |  |  | 0.685^2^ |
| Primary | 60 (56.6%) | 128 (61.5%) | 61 (58.7%) |  |
| Secondary | 46 (43.4%) | 80 (38.5%) | 43 (41.3%) |  |
| **Duration of infertility (y)** | 3.0 (2.0-5.0) | 3.0 (2.0-5.0) | 3.0 (2.0-5.0) | 0.622^1^ |
| **D3 serum FSH level (IU/L)** | 6.18 (5.12-7.14) | 6.10 (5.09-6.91) | 6.11 (5.34-6.76) | 0.775^1^ |
| **D3 serum LH level (IU/L)** | 4.92 (3.53-7.07)^c,d^ | 6.27 (4.49-9.86)^d^ | 8.97 (6.38-11.64) | < 0.001^1^ |
| **LH/FSH ratio** | 0.82 (0.61-1.14)^c,d^ | 1.10 (0.77-1.54)^d^ | 1.46 (1.06-1.96) | < 0.001^1^ |
| **Testosterone(ng/ml)** | 0.43 (0.27-0.65) | 0.36 (0.23-0.55) | 0.43 (0.28-0.56) | 0.048^1^ |
| **TSH (μIU/mL)** | 1.95 (1.36-2.74) | 2.05 (1.49-3.20) | 2.09 (1.44-3.18) | 0.394^1^ |
| **Fasting glucose (mmol/L)** | 5.41 (5.10-5.70) | 5.31 (5.05-5.66) | 5.26 (4.99-5.60) | 0.266^1^ |
| **Fasting insulin (μU/mL)** | 13.30 (8.00-20.40) | 11.90 (7.70-16.10) | 10.90 (8.35-15.70) | 0.376^1^ |
| **QUICKI** | 0.322±0.032 | 0.328±0.028 | 0.332±0.029 | 0.213^3^ |
| **Hyperandrogenemia, %** | 48 (45.3%)^e^ | 65 (31.3%) | 39 (37.5%) | 0.049^2^ |
| **Polycystic ovaries, %** | 70 (66.0%)^c,d^ | 181 (87.0%) | 99 (95.2%) | < 0.001^2^ |
| **Menstrual regularity** |  |  |  | 0.044^2^ |
| Regular periods, % | 6 (5.7%) | 6 (2.9%) | 4 (3.8%) |  |
| Oligomenorrhea, % | 92 (86.8%) | 168 (80.8%) | 78 (75.0%) |  |
| Amenorrhea, % | 8 (7.5%)^a^ | 34 (16.3%) | 22 (21.2%) |  |
| **Gonadotropin start dose(IU)** | 150.0 (150.0-225.0)^c,d^ | 150.0 (112.5-150.0)^d^ | 112.5 (112.5-150.0) | < 0.001^1^ |
| **Duration of gonadotropin stimulation (d)** | 9.0 (8.0-10.0)^b^ | 10.0 (8.0-11.0) | 10.0 (9.0-12.0) | 0.002^1^ |
| **Total dose of gonadotropin used (IU)** | 2025.0 (1528.1-2521.9)^d,f^ | 1687.5 (1284.4-2137.5)^d^ | 1350.0 (1125.0-1762.5) | < 0.001^1^ |
| **Serum E_2_ level (pg/mL) on the day of ovulatory dose of hCG** | 2434.5 (1715.0-3782.8)^a^ | 2568.5 (1968.3-3611.0) | 2897.5 (2081.3-4460.0) | 0.037^1^ |
| **Endometrial thickness (mm) on the day of ovulatory dose of hCG** | 11 (10-13) | 11 (10-12) | 11 (10-12) | 0.074^1^ |
| **No. of oocyte retrieved** | 13 (10-16) | 14 (10-17) | 14 (11-17) | 0.105^1^ |
| **No. of oocyte fertilized** | 10 (8-13) | 11 (8-14) | 11 (8-13) | 0.264^1^ |
| **No. of embryos transferred** | 2 (2-2) | 2 (2-2) | 2 (2-2) | 0.796^1^ |
| **Cycles with different technologies** | |  |  | 0.912^2^ |
| IVF | 94 (88.7%) | 188 (90.4%) | 93 (89.4%) |  |
| ICSI | 12 (11.3%) | 20 (9.6%) | 11 (10.6%) |  |
| **Embryo type** |  |  |  | 0.073^2^ |
| Cleavage embryo | 55 (51.9%) | 122 (58.7%) | 70 (67.3%) |  |
| Blastocyst | 51 (48.1%) | 86 (41.3%) | 34 (32.7%) |  |
| **Embryo quality** |  |  |  | 0.038^2^ |
| Cycle with high-quality embryos | 100 (94.3%)^e^ | 206 (99.0%) | 101 (97.1%) |  |
| Cycles without high-quality embryos | 6 (5.7%) | 2 (1.0%) | 3 (2.9%) |  |

Note: BMI=body mass index; FSH=follicle-stimulating hormone; LH=luteinizing hormone; TSH=thyroid-stimulating hormone; QUICKI=quantitative insulin sensitivity check index; E_2_=estradiol; IVF=in vitro fertilization; ICSI=intracytoplasmic sperm injection; hCG=human chorionic gonadotropin.

Values are numbers (percentages) of participants, median (interquartile range) or mean ± standard deviation.

^1^Kruskal-Wallis test followed by a post-hoc pairwise comparison.

^2^Chi-square test followed by Bonferroni post-hoc test.

^3^One-way ANOVA followed by Bonferroni post-hoc test.

^a^*P*<0.05, statistically significant differences from high-AMH group.

^b^*P*<0.01, statistically significant differences from high-AMH group.

^c^*P*<0.001, statistically significant differences from average-AMH group.

^d^*P*<0.001, statistically significant differences from high-AMH group.

^e^*P*<0.05, statistically significant differences from average-AMH group.

^f^*P*<0.01, statistically significant differences from average-AMH group.
